# Research progress on RNA−binding proteins in breast cancer

**DOI:** 10.3389/fonc.2022.974523

**Published:** 2022-08-18

**Authors:** Ying Chen, Hai Qin, Lufeng Zheng

**Affiliations:** ^1^ School of Life Science and Technology, Jiangsu Key Laboratory of Carcinogenesis and Intervention, China Pharmaceutical University, Nanjing, China; ^2^ Department of Clinical Laboratory, Guizhou Provincial Orthopedic Hospital, Guiyang, China

**Keywords:** breast cancer, RNA-binding proteins, research progress, mRNA, 3’UTR

## Abstract

Breast cancer is the most common malignancy in women and has a high incidence rate and mortality. Abnormal regulation of gene expression plays an important role in breast cancer occurrence and development. RNA-binding proteins (RBPs) are one kind of the key regulators for gene expression. By interacting with RNA, RBPs are widely involved in RNA cutting, transport, editing, intracellular localization, and translation regulation. RBPs are important during breast cancer occurrence and progression by engaging in many aspects, like proliferation, migration, invasion, and stemness. Therefore, comprehensively understanding the role of RBPs in breast cancer progression can facilitate early diagnosis, timely treatment, and long-term survival and quality of life of breast cancer patients.

## Introduction

Breast cancer is the most common malignancy affecting women and is a highly heterogeneous disease including several subtypes, which are defined by the differential expression of receptors on the cell surface (1). The progression and occurrence of breast cancer are contributed by the ectopic gene expression, which is regulated transcriptionally, post-transcriptionally, translationally, and post-translationally.

**Figure 1:**
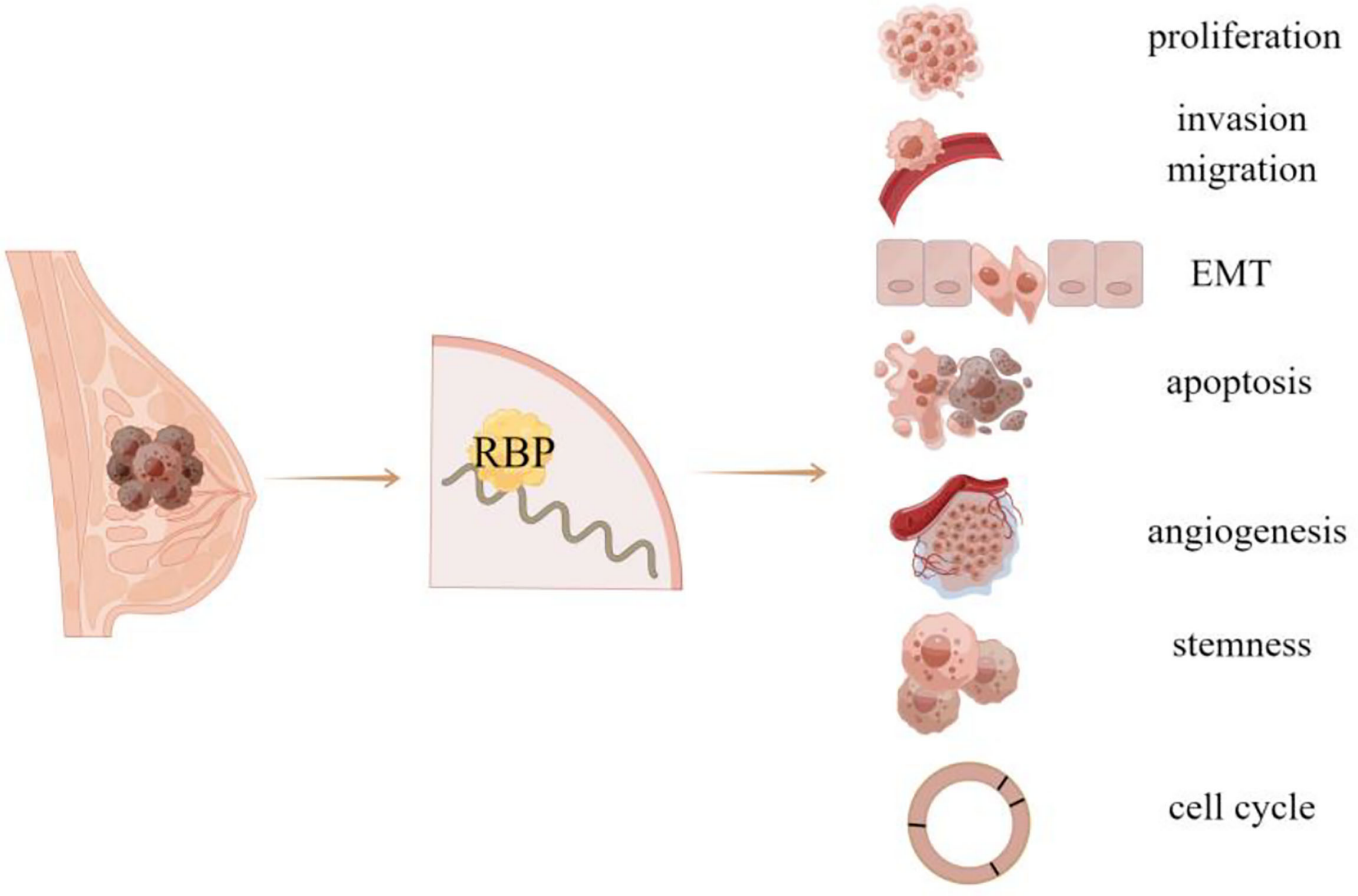
RBP is related to the occurrence and progression of breast cancer.

RNA biosynthesis and metabolism is one of the key steps of gene regulation. An increasing number of evidence shows that RNA expression profile in cancer cells is significantly different from that in benign cells, suggesting that the abnormal regulation of RNA metabolism may be related to the occurrence and progression of tumors (2). RNA binding proteins (RBPs) are the major regulators of RNA metabolism and crucial in all steps of gene expression (3). As a kind of unstable and degradable biomacromolecules, mRNAs bind to specific RBPs and form complexes to maintain their stability in cells, within which RBPs control the localization, stability, translation, and degradation of RNA by binding to different regions of mRNAs . In addition, the binding of RBPs to RNA contributes to RNA metabolism at different stages and regulates its subsequent function. Currently, exceeding 2000 RBPs are known to interact with RNA through different binding surfaces. The roles of abnormally expressed RBPs in human diseases (such as cancer, viral infection) and the potential application of RBPs as a therapeutic target or diagnostic marker represent a rapidly expanding research field (4). RBPs are disordered in various tumors, and affect the expression and function of tumor-related transcripts, so as to play different biological roles in tumor progression, such as proliferation, invasion, migration, stemness, and angiogenesis. This article will review recent advances in RBPs related to breast cancer (**Figure 1** **;** **Table 1**).

**Table 1:** Summary of the cellular functions of RBPs in breast cancer.

RBP	Expression in breast cancer	Functions	Pathways/targets	References
RBM38	Downregulation	Inhibits proliferation, invasion, migration, EMT; regulates the cell cycle	p53, c-Myc, PTEN, ZO-1, STARD13-correlated ceRNA network	(5–9)
PCBP2	Upregulation	Promotes migration, proliferation, invasion, stemness, EMT and cholesterol synthesis; inhibits apoptosis	UFD1, NT5E, lnc030, SQLE, PI3K/Akt signaling pathways	(10, 11)
QKI	Downregulation	Inhibits self-renewal, EMT, cell contact, proliferation, migration, invasion; regulates the cell cycle and apoptosis	RASA1, MAPK signaling pathways, FOXO1, lncRNA ST8SIA6-AS1	(12–14)
HuR	Upregulation	Promotes invasion, proliferation, migration, angiogenesis; regulates the cell cycle; inhibits apoptosis	Snail, MMP-9, uPAR, FOXQ1, VEGFA, CDK3, lncRNA AGAP2-AS1, MTA1, TNF-α	(15–23)
LIN28	Upregulation	Promotes proliferation, migration, invasion, stemness; regulates aerobic glycolysis, Warburg effect and pH	let-7, CAIX, miR-638, CREB1, VASP, MSI2, YAP1, Hippo signaling pathways	(24–32)
SAM68	Upregulation	Promotes survival, proliferation, migration and invasion; regulates the cell cycle	CBP/β-catenin, Insulin and leptin signaling pathway, MAPK/PI3K signaling pathways, p21 and p27, FOXO, Akt/GSK-3β signal transduction, Rad51, PARP	(33–38)
MSI	Upregulation	Promotes stemness, chemoresistance and proliferation; regulates the cell cycle; inhibits apoptosis and invasion	p21^Cip1^, TAC1, EMT, ERK1/2, TP53INP1, ESR1, Notch	(39–47)

## RNA-binding motif protein 38 (RBM38)

RBM38, also known as RNPC1, is a member of the RBP family. *RBM38* gene is located on chromosome 20q13 and belongs to the RNA recognition motif (RRM) family of RBP. *RBM38* contains the classical RRM domain and is expressed as RNPC1a with 239 amino acids and RNPC1b with 121 amino acids, respectively. RBM38 exists in various tissues (48), including bone marrow, lymph nodes, human blood, brain, breast, colorectal, lung, and other organs. Additionally, RBM38 has been shown to participate in the progression of breast cancer (49), acute myeloid leukemia (50), colorectal cancer, and renal cell carcinoma (51), *via* transcriptionally regulating many downstream targets in different ways (51).

Studies have shown that ectopic expression of RBM38 can inhibit the proliferation of breast cancer cells, while knockdown of RBM38 exhibits an opposite effect *in vivo* and *in vitro* (52). When RBM38 is overexpressed, it inhibits the migration and invasion of breast cancer cells by inducing cell cycle arrest and inhibiting mutant p53-induced epithelial-mesenchymal transition (EMT) (5). EMT is triggered by individual extracellular signals, including extracellular matrix components, such as collagen and hyaluronic acid, and soluble growth factors, like transforming growth factor–β (TGF-β), fibroblast growth factor (FGF), and epidermal growth factor (EGF). TGF-β is one of the most famous EMT inducers (53). Elevated levels of TGF-β in malignant breast cells enhance breast cancer invasion, migration, and immune evasion. It is found that TGF-β induces a significant down-regulation of RBM38 in breast cancer, which is directly regulated by Snail, a transcription factor targeting at the E-box element of the *RBM38* gene promoter region (6). In addition, Zonula occludens-1 (ZO-1) is downregulated in response to TGF-β, which can control endothelial cell-cell tension, cell migration, and barrier formation, while RBM38 positively regulates ZO-1 transcript by directly binding to AU/U rich elements (Ares) on its mRNA 3’-untranslated region (3’UTR), thus inhibiting cell migration and invasion (6).

**Figure 2:**
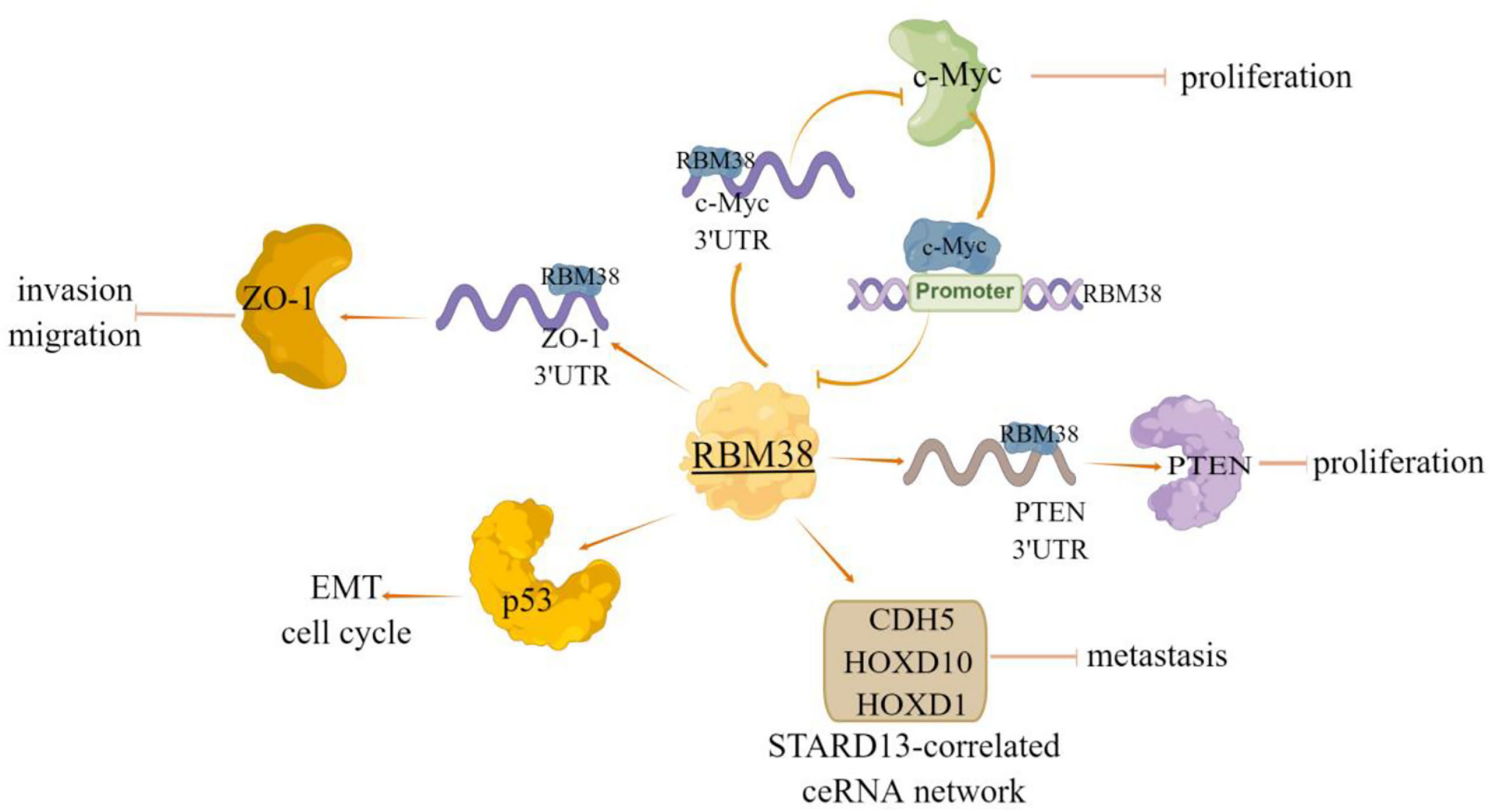
The regulatory mechanism of RBM38 in breast cancer. RBM38 can directly bind 3’UTR of *ZO-1* and *PTEN*, positively regulate their transcripts, and inhibit cell migration and invasion. RBM38 inhibits the migration and invasion of breast cancer cells by inducing cell cycle arrest and inhibiting mutant p53-induced EMT. RBM38 can act as a tumor suppressor by forming a regulatory loop with related genes, and inhibit c-Myc expression by directly targeting 3’UTR of *c-Myc* mRNA. In turn, c-Myc inhibits RBM38 expression by directly binding to the E-box motif in the *RBM38* promoter region. RBM38 can promote ceRNA interactions among STARD13, CDH5, HOXD10 and HOXD1 (STARD13-correlated ceRNA network), by promoting the expression of these four genes, inhibit breast cancer cell metastasis. Arrows indicate activation and blunted lines indicate inhibition.

Furthermore, RBM38 often functions by forming regulatory loops with related genes, for example, Li et al. (7) have shown that RBM38 acted as a tumor suppressor by inhibiting the expression of c-Myc *via* directly targeting the Ares in *c-Myc* mRNA 3’UTR, and thus destabilizing *c-Myc* transcript; In turn, c-Myc inhibits RBM38 expression by directly binding to the E-box motif in the promoter region of *RBM38* gene. RBM38 can also function through the tumor suppressor PTEN, as evident by that RBM38 can enhance the stability of PTEN mRNA and increase the expression of PTEN protein by directly targeting *PTEN* 3’UTR (8). Notably, our previous study also identified novel targets of RBM38 in breast cancer. We found that the expression of RBM38 was positively correlated with the relapse free survival and overall survival of patients with breast cancer, and RBM38 can promote the competing endogenous RNA (ceRNA) network crosstalk among STARD13, CDH5, HOXD10 and HOXD1 (STARD13-correlated ceRNA network) and then inhibit the metastasis of breast cancer cells (9) (**Figure 2**). Zhu et al. (54) selected 161 cases of breast cancer tissues to explore the relationship between RBM38 expression and distant metastasis and prognosis of breast cancer. The results showed that the high expression of RBM38 was positively correlated with the low rate of distant metastasis and good prognosis in patients with breast cancer **(** **Table 2** **)**.

**Table 2:** RBP and clinical relevance.

RBP	Clinical Relevance	References
RBM38	The high expression of RBM38 is positively correlated with the low rate of distant metastasis and good prognosis in patients with breast cancer.	(54)
HuR	Patients with high levels of cytoplasmic HuR have a higher risk of metastasis.	(17)
LIN28	The expression of LIN28 is related to the stage and subtype of advanced disease in patients with breast cancer, and the expression of LIN28 may be an independent prognostic factor.	(30)
MSI	MSI-1 is a negative prognostic marker for disease-free and distant metastasis free survival of breast cancer, which has a negative impact on the overall survival rate. Low expression of MSI-2 is associated with poor prognosis in patients with breast cancer.	(40, 42)

## Poly (C) binding protein 2 (PCBP2)

**Figure 3:**
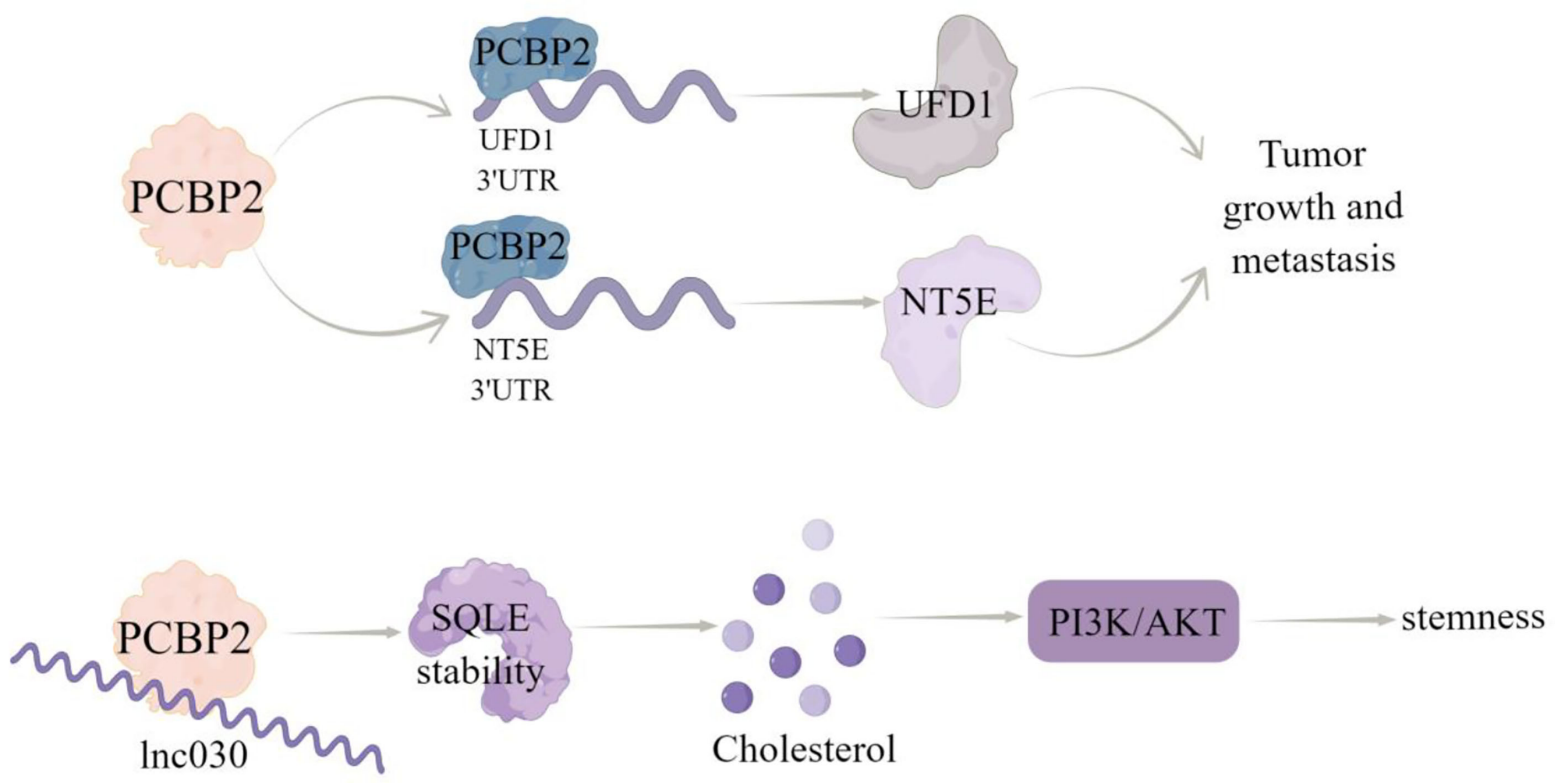
The regulatory mechanism of PCBP2 in breast cancer. PCBP2 can directly bind 3’UTR of UFD1 and NT5E, positively regulate their transcripts,and promote tumor growth and metastasis. Lnc030 cooperates with PCBP2 to stabilize SQLE mRNA, increase cholesterol synthesis, activate PI3K/Akt signal transduction, and regulate cholesterol synthesis and stemness properties of BCSCs. Arrows indicate activation and blunted lines indicate inhibition.

PCBP family plays a central role in transcriptional and translational regulation, including mRNA stability, translation silencing, and translation enhancement (55–57). It has been proved that PCBP plays an important role in tumor progression, including apoptosis, proliferation, invasion, and EMT (58). PCBP2, a member of the PCBP family, is an RBP that can regulate RNA stability *via* directly binding to the single stranded poly (C) motifs of RNAs (10). Several studies have demonstrated the functional role of PCBP2 in the progression of several cancers, including glioma, gastric cancer, pancreatic ductal adenocarcinoma, and hepatocellular carcinoma (58–61). In breast cancer, Thomas et al. (62) used RNA sequencing data to analyze the expression patterns of transcriptional subtypes of eleven estrogen receptor positive (ER+) subtypes and fourteen triple negative (TN) subtypes of breast tumors compared the RNAseq data of 594 cases from the TCGA cohort and identified several RNA processing factors differentially expressing among tumor subtypes and/or regulated by ER, among which *PCBP2* was ranked. A recent study showed that the expression of PCBP2 protein was increased significantly in breast cancer tissues and cell lines, which was due to selective cleavage and polyadenylation (APA) (10). Functionally, PCBP2 promoted the carcinogenesis and metastasis of breast cancer by directly regulating the expression of ubiquitin recognition factor in ER-associated degradation 1 (UFD1) and 5’-nucleotidase ecto (NT5E) *via* binding to their 3’UTRs (10). Additionally, Qin et al. (11) identified that a new long non-coding RNA (lncRNA), named lnc030, is highly expressed in breast cancer stem cells (BCSCs) *in vitro* and *in vivo*. And lnc030 cooperates with PCBP2 to stabilize squalene cyclooxygenase (SQLE) mRNA, resulting in increased cholesterol synthesis, which subsequently facilitates the stemness of BCSCs *via* activating PI3K/Akt signaling (**Figure 3**).

## Quaking (QKI)

QKI belongs to the RBP of signal transduction and activation RNA (STAR) family (63). It was firstly found in and important for the central and peripheral nervous systems. Human *QKI* gene expresses three main alternative splicing transcripts, namely *QKI-5*, *QKI-6* and *QKI-7*, among which QKI-5 is the only subtype located in the nucleus, while QKI-6 and QKI-7 are distributed in the cytoplasm (64). QKI specifically binds to cis elements in 3’UTR (65), and regulates precursor RNA (pre-mRNA) processing, mRNA output, mRNA stability, and protein translation of target genes (66–69). Increasing evidence showed that QKI may be a tumor suppressor in various malignant tumors, including colon cancer, lung cancer, oral cancer, and prostate cancer (70–73), for example, QKI impairs the self-renewal and EMT of oral squamous cell carcinoma cells (70–73); QKI can also regulate cell communication and inhibit the progression of clear cell renal cell carcinoma (74). Notably, QKI mRNA and protein are downregulated in breast cancer and decreased QKI expression was significantly associated with ER, PR, and HER2 positive, non-basal-like breast carcinoma and non-triple-negative breast cancer. Meanwhile, QKI expression is positively correlated with the survival of patients, suggesting the prognostic value of QKI in breast cancer patients (12).

Cao et al. (12) have shown that the decreased expression or activity of RASA1 can activate MAPK signaling pathway by reducing the GTPase activity of Ras protein, thereby increasing cell proliferation, inhibiting apoptosis and regulate cell cycle distribution; QKI can directly bind to RASA1 mRNA and enhance RASA1 expression by stabilizing its transcripts, through which QKI overexpression weakens the phosphorylation of MAPK signaling pathway, thereby inhibiting the activation of MAPK pathway and breast cancer progression. In addition, Forkhead box O1 (FoxO1) is a key tumor suppressor for cell proliferation, which can control cell cycle and apoptosis and dysregulation of *FoxO1* expression has been observed in various cancers (13). QKI may cause dysgenesis of FoxO1 through post-transcriptional inhibition, and lead to the occurrence and progression of breast cancer, which is characterized by that QKI can directly bind to the 3’UTR of *FoxO1* and reduce its mRNA stability, this effect is critical for breast cancer occurrence and progression (13).

**Figure 4:**
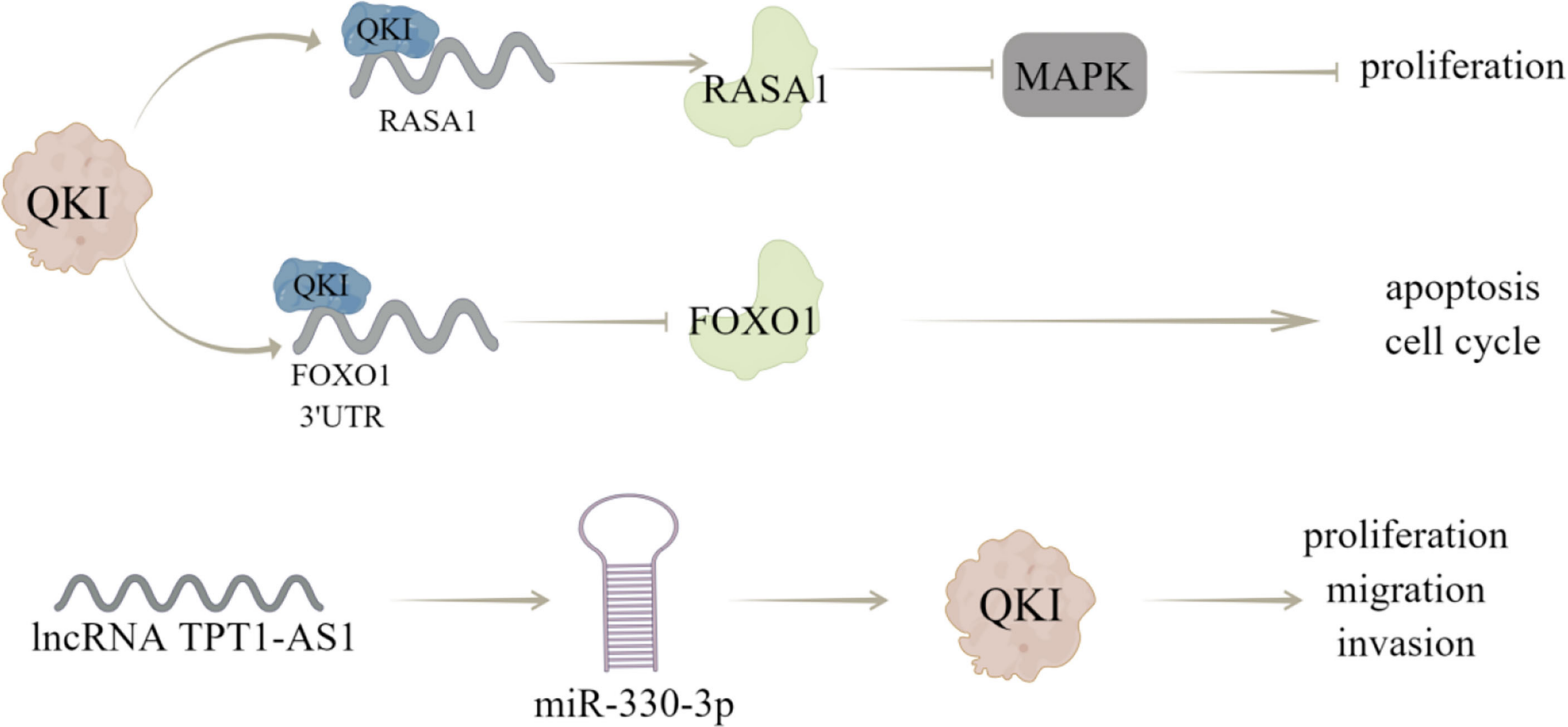
The regulatory mechanism of QKI in breast cancer. QKI can directly combine with *RASA1* mRNA to enhance its expression and reduce the phosphorylation of MAPK signaling pathway, thus inhibiting the activation of MAPK pathway and proliferation of breast cancer cells. QKI can directly bind to 3’UTR of *FoxO1*, reduce its mRNA stability, and regulate cell cycle and apoptosis. LncRNA *TPT1-AS1* can act as a ceRNA of *miR-330-3p* to up regulate QKI expression, thus inhibiting the proliferation, migration, and invasion of breast cancer cells. Arrows indicate activation and blunted lines indicate inhibition.

Furthermore, QKI can bind to ncRNAs to modulate breast cancer progression, like Chen et al. (75) demonstrated that QKI could interact with lncRNA *ST8SIA6-AS1*, to promote proliferation, migration, and invasion of breast cancer cells. And Hu et al. (14) showed that lncRNA *TPT1-AS1* may act as a ceRNA of *miR-330-3p* to upregulate QKI expression, thereby inhibiting the proliferation, migration, and invasion of breast cancer cells. Notably, Gu et al. (76) carried out immunohistochemical evaluation (IHC) of QKI protein expression and prognostic value of 108 patients with breast cancer. The obtained results showed that QKI expression predicted a better prognostic value in BC patients, and the correlation between QKI and EMT was verified in the coexpression analysis of METABRIC datasets (**Figure 4**).

## Hu-antigen R (HuR)

HuR, also known as embryonic lethal abnormal visual protein 1 (ELAVL1), is a

widely expressed post-transcriptional regulatory factor. Although HuR is mainly located in the nucleus, its function of stabilizing and regulating target mRNA translation is tightly related to its translocation to the cytoplasm (77). HuR preferentially binds to mRNA with adenine and uridine rich elements (ARE) or uridine rich sequences, usually locating in 3’UTR (78, 79). ARE is a specific cis element of mRNA, which can target mRNAs for rapid exosomal degradation (80).

HuR has been reported to interact with the mRNA 3’UTR of transcription factor Snail, metallopeptidase MMP-9 (15), and serine protease uPAR (16), among which Snail can induce EMT, while MMP-9 and uPAR are involved in extracellular matrix (ECM) degradation. Therefore, HuR is thought to promote invasion and metastasis by increasing the expression of proteins that induce EMT and degrade ECM. In consistent, inhibition of HuR using the small molecule inhibitor KH-3, can inhibit the invasion of breast cancer cells by destroying the HuR-FOXQ1 mRNA interaction (17). Additionally, Yang et al. (18) proved that HuR protein may be a useful target for the screening of anti-tumor angiogenesis drugs, as they found that a compound ZM-32 could effectively prevent the formation of HuR RRM1/2–VEGFA mRNA complex, thus suppressing the proliferation, migration, growth, and angiogenesis of breast cancer cells. Zhu et al. (19) also revealed the HuR-dependent anti-angiogenic effect of eltrombopag in breast cancer, and further emphasized the effectiveness of HuR inhibitors on tumor inhibition, especially angiogenesis.

**Figure 5:**
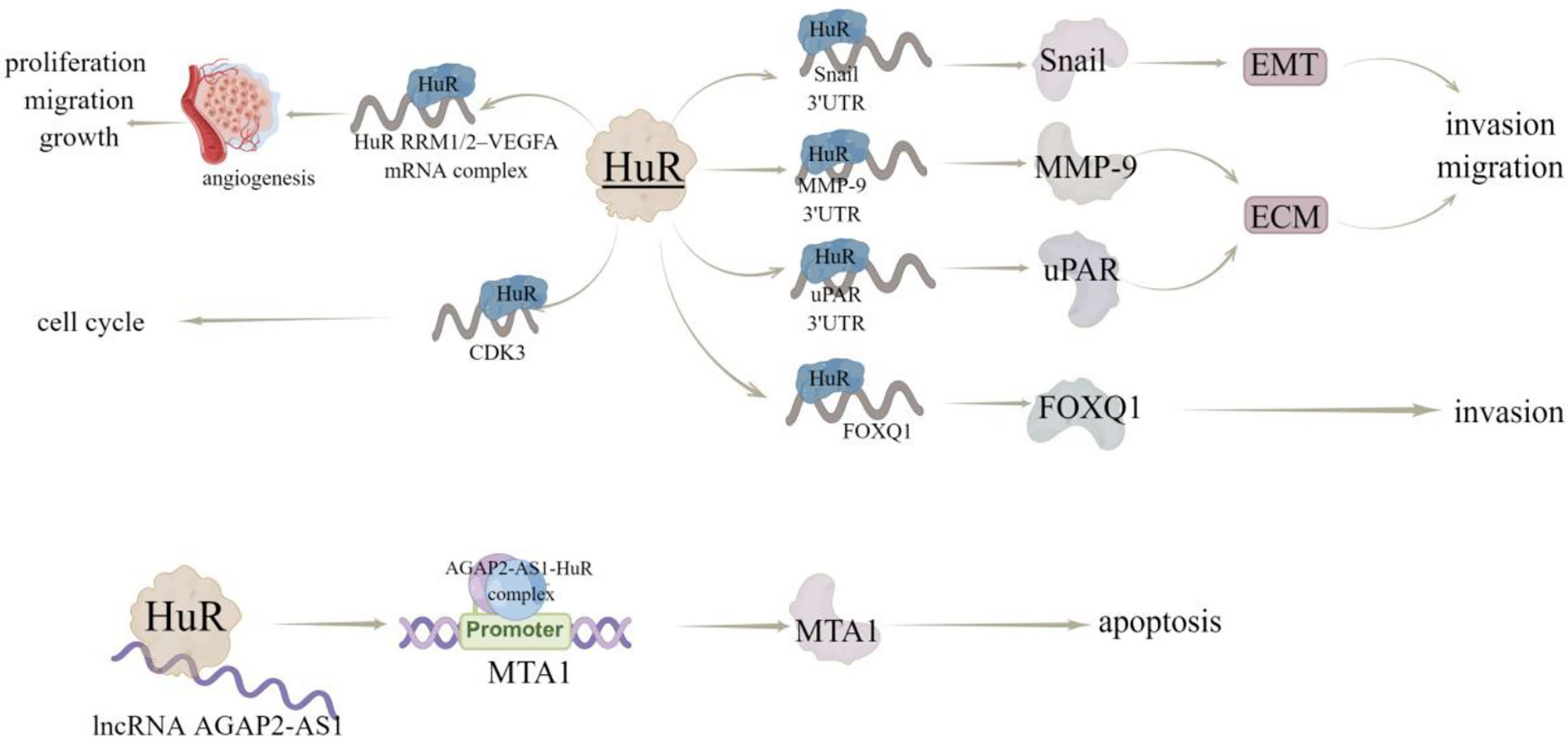
The regulatory mechanism of HuR in breast cancer. HuR interacts with 3’UTR of *Snail*, *MMP-9* and *uPAR*, regulates EMT and EMC, and promotes invasion and metastasis. HuR interacts with *FOXQ1* mRNA to inhibit the invasion of breast cancer cells. HuR can form a complex with RRM1/2–VEGFA mRNA to promote tumor growth and angiogenesis. Hur can bind to lncRNA *AGAP2-AS1* to stabilize AGAP2-AS1 expression. AGAP2-AS1-HuR complex up regulates H3K27ac level in *MTA1* promoter region to increase *MTA1* promoter activity and MTA1 expression, thus enhancing resistance to apoptosis. Arrows indicate activation and blunted lines indicate inhibition.

Cyclin dependent kinase (CDK) plays a key role in regulating the process of cell cycle. In human cancers, including breast cancer, hepatocellular carcinoma and lymphoma, a series of upstream regulatory factors and downstream substrates of CDK participate in abnormal CDK-related signal transduction (20). HuR can directly bind to and regulate the expression of CDK3 mRNA, thereby promoting the progression of breast cancer (21). In addition, HuR and CDK3 expression levels were positively correlated and significantly up-regulated in breast cancer samples (21). Furthermore, Wang et al. (22) revealed that docetaxel (DTX) induces apoptosis of MCF-7 cells through the SIDT2/NOX4/JNK/HuR axis-mediated TNF-α expression. Notably, HuR can also bind to ncRNA in breast cancer, like Wu et al. (23) determined that HuR could bind to lncRNA *AGAP2-AS1* to stabilize AGAP2-AS1 expression and the AGAP2-AS1-HuR complex upregulates H3K27ac level in *MTA1* promoter region to improve *MTA1* promoter activity and expression, thereby inducing the resistance breast cancer cells to apoptosis (**Figure 5**). Wu et al. (17) studied 140 samples of patients with breast cancer and found that high cytoplasmic HuR was significantly correlated with high tumor grade, low overall survival and distant disease-free survival. In addition, in the sample, 63.6% of patients with metastasis have high cytoplasmic HuR, indicating that patients with high levels of cytoplasmic HuR have a higher risk of metastasis **(** **Table 2** **)**.

Based on the critical roles of HuR in cancer cells, a variety of methods have been developed to inhibit HuR, including inhibiting HuR/mRNA interaction, HuR dimerization/polymerization, HuR nuclear/cytoplasmic shuttle, and HuR expression (81). Meanwhile, some inhibitors of HuR were identified, including MS-444, which can block HuR dimerization and nuclear/cytoplasmic shuttle (82); CMLD2, dihydrotanshinone-I (DHTs) and suramin can regulate the interaction of HuR/mRNA (83–85); Okicenone, trichostatin, 5-aza-2’-deoxycytidine (AZA) can also be used as inhibitors of HuR shuttle (82, 86). Recently, based on the fact that the function of HuR in cancer cells depends on its dimerization and its nuclear/cytoplasmic shuttle, Natalia et al. (81) identified a new kind of HuR polymerization inhibitor SRI-42127, which can inhibit the formation of HuR polymers in glioblastoma exotoxin (PDGx) from primary patients, resulting in proliferation arrest, induction of apoptosis and inhibition of colony formation.

## LIN28

LIN28, a highly conserved RBP, has two homologues LIN28A/B in mammals (87) and expressed in various human epithelial tumors, such as lung cancer (88), ovarian cancer (89), hepatocellular carcinoma (90), and colorectal cancer (91). LIN28 is a member of a reprogramming factor that interacts with KLF4, SOX2, and NANOG to induce pluripotency in adult fibroblasts (91). LIN28 can block the production of let-7 miRNA and subsequently disinhibit let-7 miRNA target genes (*RAS*, *myc* and *HMGA2*), which plays an important role in CSC maintenance (92). In consistent, LIN28 is highly enriched in BCSC population and plays an important role in maintaining CSC characteristics (92). Notably, let-7 miRNA family has been identified as a downstream target of LIN28 to inhibit let-7 maturation. The blocking of let-7 biogenesis and subsequent dis-inhibition of let-7 target genes by LIN28 has been proved to be the potential mechanism contributing to LIN28-induced cancer progression and metastasis (93). All let-7 family members are regulated by LIN28 *via* blocking its processing into mature miRNA, conversely LIN28 is also down-regulated by let-7, forming a regulatory-loop (94).

Additionally, abnormal expression of LIN28 and let-7 promotes aerobic glycolysis or Warburg effect in cancer cells (95). Carbonic anhydrase IX (CAIX) is a hypoxia induced transmembrane protein that catalyzes the reversible hydration of carbon dioxide to bicarbonate ions and protons (25). It contributes to the neutralization of intracellular pH, plays a vital role in maintaining favorable intracellular pH (PHI), provides selective advantages for cancer cells and promotes cancer progression (26). In hypoxia breast cancer cell lines, inhibition of CAIX affects let-7/LIN28 axis, thereby affecting the related metabolic pathways and stem cell reprogramming (27). Vasodilator stimulated phosphoprotein (VASP) is an important cytoskeleton related protein belonging to Ena/VASP protein family (28). VASP is a key target protein that regulates the migration of various tumor cells and upregulated in breast cancer tissues and cells (29). VASP silencing inhibits the invasion of breast cancer MDA-MB-231 cells, and *miR-638* can inhibit the expression of VASP (29). LIN28 can also regulate the processing of *miR-638*, thus inhibiting its maturation and promoting VASP expression, while CREB1, as a transcription factor, binds to the promoter of *LIN28* gene and activates the LIN28/miR-638/VASP pathway, which promotes the proliferation and migration of breast cancer cells. Xu et al. (30) collected data from 291 patients with breast cancer, in which the expression level of LIN28 was evaluated by immunohistochemical staining. The found that the positive expression of LIN28 is related to lymph node metastasis, HER-2, estrogen receptor and progesterone receptor, and Kaplan-Meier analysis showed that the overall survival rate of LIN28 positive patients was lower than that of LIN28 negative patients. These data suggest that the expression of LIN28 is related to the stage and subtype of advanced disease in patients with breast cancer, and the expression of LIN28 may be an independent prognostic factor **(** **Table 2** **)**.

Notably, LIN28 can also act as a transcription or translation regulator independent of let-7 (31). LIN28 recruit RNA binding protein Musashi-2 (MSI-2) by LIN28 CSD domain and MSI2 RRM domain, which directly induces the mRNA decay of YAP1 upstream kinase and negatively regulates Hippo pathway, resulting in the activation of YAP1, enhancing CSC like characteristics, tumorigenesis and metastasis in TNBC cells (32).

**Figure 6:**
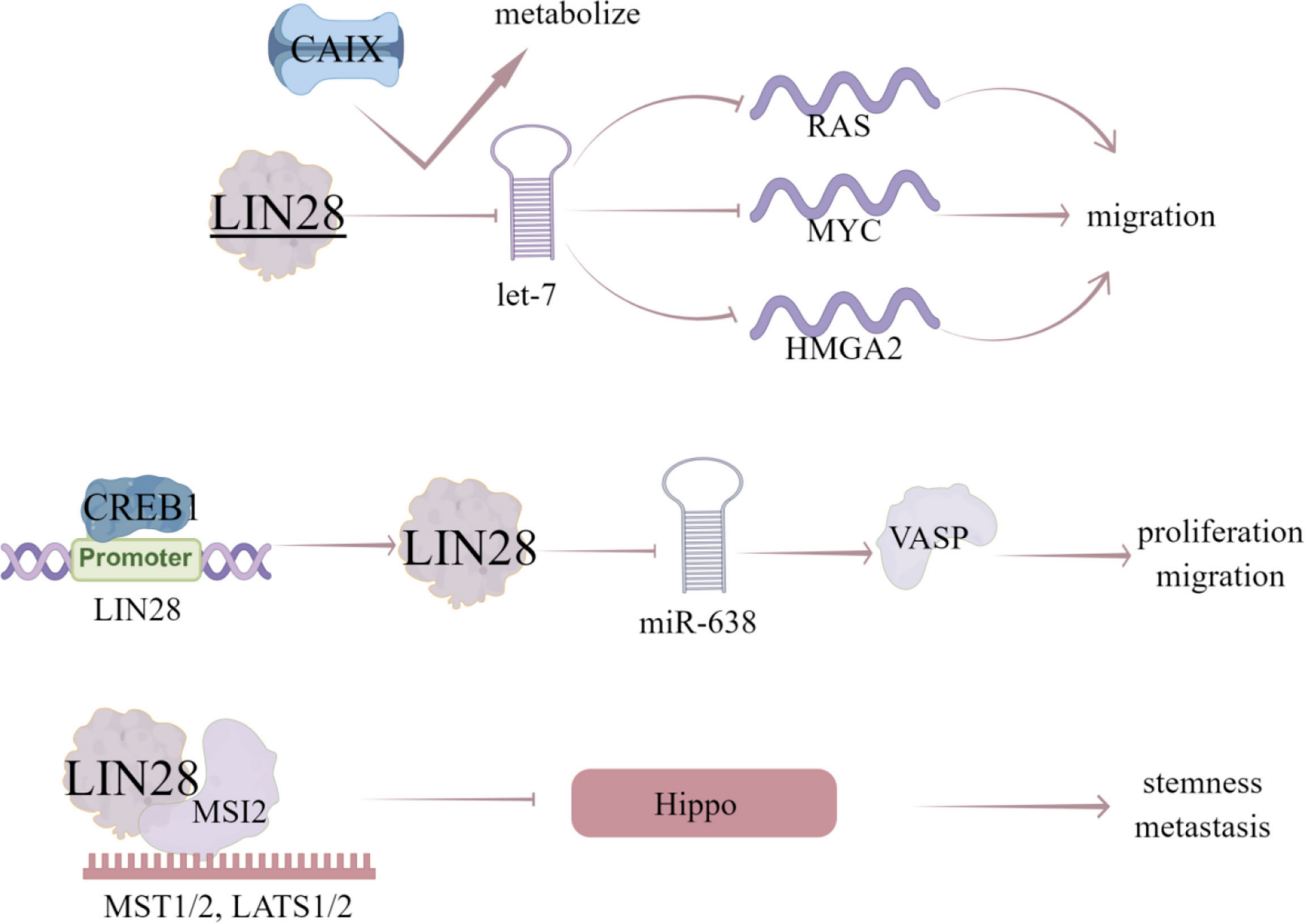
The regulatory mechanism of LIN28 in breast cancer. LIN28 can block the production of *let-7* miRNA and disinhibit the target genes of *let-7* miRNA (*RAS, MYC* and *HMGA2*), thus promoting the migration of breast cancer cells. Inhibition of CAIX will affect let-7/LIN28 axis and related metabolic pathways. CREB1 binds to the promoter of *LIN28*, activates LIN28/miR-638/VASP pathway, and promotes the proliferation and migration of breast cancer cells. LIN28 can recruit MSI2, directly induce the mRNA decay of upstream kinase of YAP1, and negatively regulate Hippo pathway, leading to the activation of YAP1, thus enhancing CSC like characteristics, tumorigenesis and metastasis in TNBC cells. Arrows indicate activation and blunted lines indicate inhibition.

Based on the let-7/LIN28 axis, researchers have found a series of inhibitors of LIN28, such as tetrahydroquinoline (THQ) -containing Povarov scaffolds. By changing the substituents of 2-Benzoic acid, fused rings at positions 3 and 4, and the substituents of phenyl part of tetrahydroquinoline core, the structure of THQ molecule has been optimized, which can be used as inhibitors to destroy the protein-RNA interaction of LIN28-let-7 (96). Trisubstituted pyrrolidone can also act as a small molecule inhibitor to destroy the protein-RNA interaction between Lin28 and let-7 (97). In addition, C1632 is a small molecule inhibitor of LIN28, which can increase let-7 level, reduce PD-L1 and inhibit a variety of cell growth (97). Furthermore, the inhibitor TPEN can make the zinc finger domain of LIN28 unstable (98) (**Figure 6**).

## SAM68

SAM68, also known as KHDRBS1, belongs to the RBP of the STAR family. It acts as a downstream target of Src family kinases in cell cycle, transcriptional regulation, cell survival and apoptosis (99). SAM68 is engaged in the progression of numerous cancers, such as MLL fusion induced leukemia (100), prostate cancer (101), breast cancer (2, 33), colon (102), and renal tumor (34).

**Figure 7:**
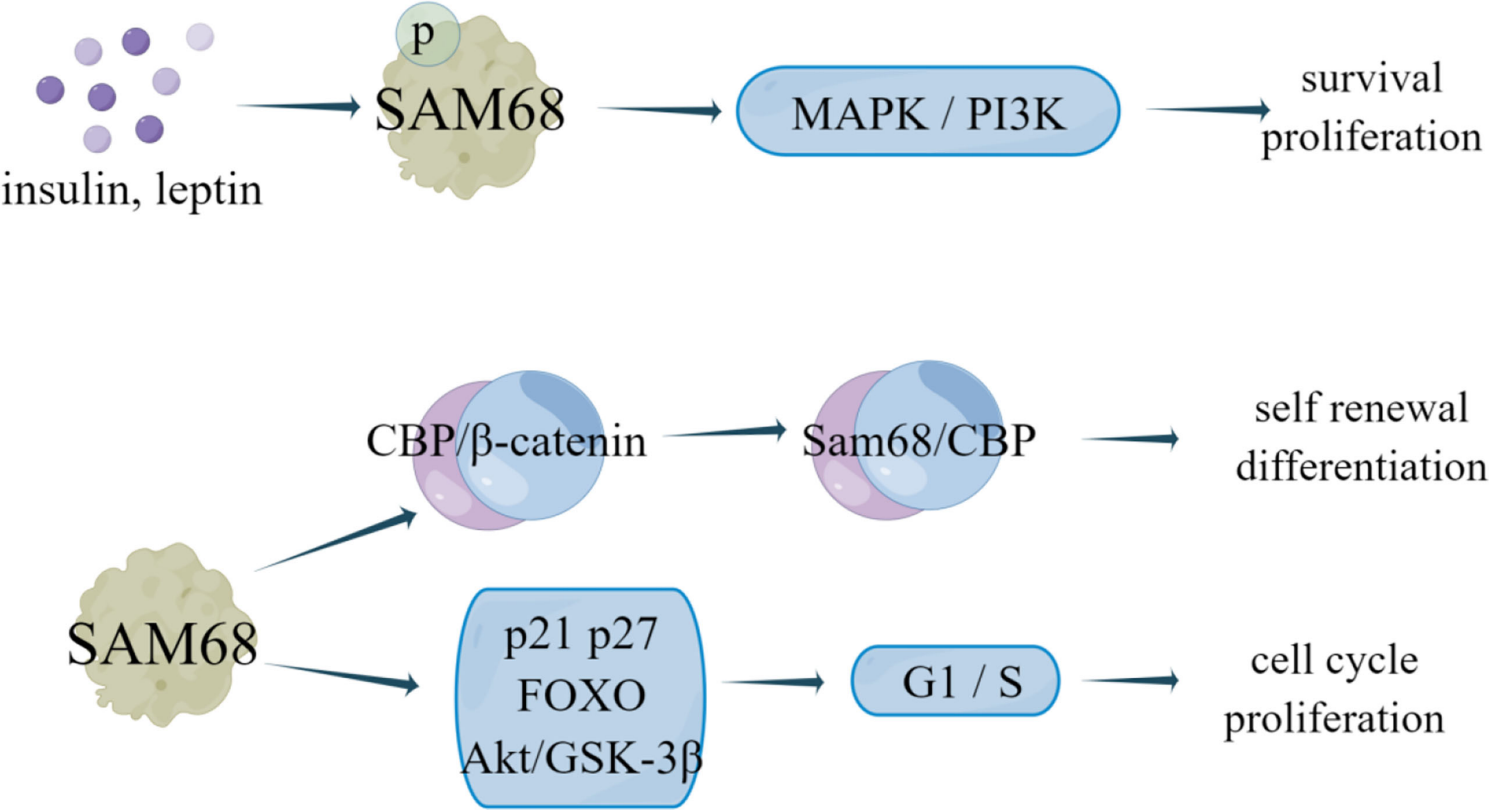
The regulatory mechanism of SAM68 in breast cancer. Insulin and leptin stimulation can promote SAM68 tyrosine phosphorylation, activate MAPK and PI3K signaling pathways in cancer, and promote cell proliferation and survival. SAM68 can form CBP-SAM68 complex in CSC, reduces CSC self-renewal and induces differentiation. Knockdown of endogenous SAM68 can inhibit cell proliferation and tumorigenicity of breast cancer cells by blocking G1 phase transition to S phase.

Yannick et al. (35) revealed that SAM68 forms a CBP-SAM68 complex in CSC, which compromises histone acetylation of known β-catenin target genes to reduce the self-renewal and induce differentiation in CSC. SAM68 can be recruited into insulin and leptin signaling pathways to mediate its effects on the survival, growth, and proliferation of different cell types. In human breast cancer cell lines MCF7, MDA-MB-231 and BT-474, the number and expression of SAM68 protein was increased under leptin or insulin stimulation, and insulin and leptin can stimulate SAM68 tyrosine phosphorylation (36). Leptin and insulin have been proven to activate MAPK and PI3K signaling pathways in cancer to promote proliferation, cell survival and cell growth (37, 38). Therefore, SAM68 not only mediates cell metabolism stimulated by insulin and leptin, but also participates in leptin- and insulin-dependent activation of MAPK and PI3K signaling pathways in breast cancer cells (36). Additionally, knockdown of endogenous *SAM68* can inhibit cell proliferation and tumorigenicity of breast cancer cells by blocking G1 phase transition to S phase, which is related to upregulation of cyclin dependent kinase inhibitor p21 Cip1 and p27 Kip1, enhanced transactivation of FOXO factor and decreased Akt/GSK-3β signal transduction (2). Notably, Alice et al. (103) showed that all molecular subtypes of breast cancer contain a subset of anti-therapeutic cells, which express high levels of Myc, Sam68 and Rad51, effectively inhibit cell survival. Analysis of a group of breast cancer patients showed that Sam68 was an independent negative factor associated with disease progression. Rad51 targeting significantly reduced the activity of Sam68-silenced breast cancer sphere cells (BCSphCs), and SAM68 is necessary as a coactivator of PARP and a synthetic lethal partner of Rad51 (103). This Sam68-PARP axis can play an important role in controlling the resistance of ER+ cells to endocrine therapy (**Figure 7**).

## Musashi (MSI)

The MSI RNA binding protein family, including two homologues Musashi-1 (MSI-1) and MSI-2, usually regulates mRNA translation and engages in tumorigenesis (104). There are two ribonucleoprotein like RNA-binding domains (RBD) in MSI protein, namely RBD1 and RBD2, which bind single stranded RNA motifs with a central UAG trinucleotide with high affinity and specificity (39). Increasing evidences indicated that MSI protein modulates the initiation and progression of various cancer cells, including lung cancer, colorectal cancer, leukemia, glioblastoma, pancreatic cancer, as well as breast cancer (39).

MSI-1, as a prognostic marker in breast cancer, has been identified as a key participant in a variety of malignancies (40). The researchers analyzed the prognostic correlation of MSI-1 with multiple survival results and found that MSI-1 is a negative prognostic marker for disease-free and distant metastasis free survival of breast cancer (40). Expression of specific breast cancer stem cells (BCSCs) is seen in aggressive tumors and MSI-1 has been shown to be one of the BCSC-related genes (41). The authors also found that silencing MSI-1 results in down-regulation of stem cell gene expression and up-regulation of cell cycle and apoptosis regulator p21 (40). Additionally, MSI-1 could also promote Notch signaling, which is a critical signaling pathway to maintain stem cell state, by binding to the mRNA of Numb, the negative regulator of Notch signaling (42). Notably, MSI-1 can downregulate the 26S proteasome by binding to the mRNA of NF-YA, the transcriptional factor regulating 26S proteasome subunit expression, thus providing an additional route by which the degradation of Notch-ICD is prevented and Notch signaling is sustained in BCSCs (43). Furthermore, loss of MSI-1 expression resulted in decreased proliferation and treatment resistance of breast cancer cells and increased apoptosis by competitively binding to tachykinin (TAC1) mRNA with miR-130a and miR-206 to stabilize and increase its translation (44).

**Figure 8:**
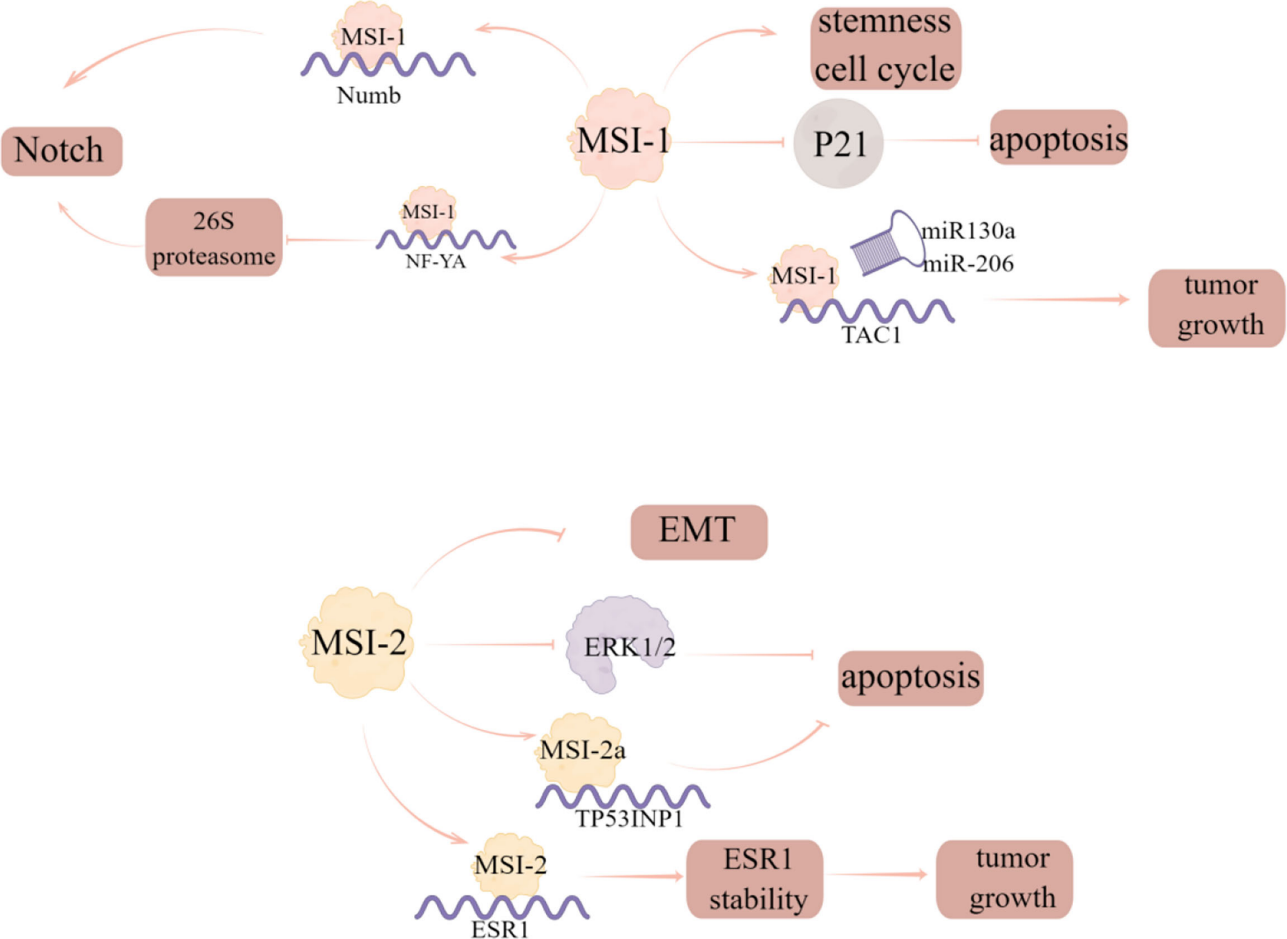
The regulatory mechanism of MSI in breast cancer. Silencing MSI-1 results in down-regulation of stem cell gene expression and up-regulation of cell cycle and apoptosis regulator p21. MSI1 competes with miR130a and -206 for interaction with TAC1 mRNA to stabilize and increase its translation and increase tumor growth. MSI-1 can promote Notch signal by binding to the mRNA of the negative regulator of Notch signal, numb, and can also down regulate 26S proteasome by binding to the mRNA of NF-YA to prevent the degradation of Notch ICD. MSI2 can inhibit EMT progression in breast cancer, and MSI2a expression inhibits TNBC invasion by stabilizing TP53INP1 mRNA and inhibiting ERK1/2 activity. MSI2 directly regulates ESR1 and affects the growth of breast cancer cells.

MSI2 is related to tumorigenesis and tumor progression of some human cancers. It is also reported that MSI2 can also inhibit the progress of EMT in breast cancer, and the low expression of MSI2 is related to the poor prognosis of breast cancer patients (45). Li et al. (46) investigated the expression and phenotypic function of two major alternative splicing MSI2 isoforms (MSI2a and MSI2b) and showed that MSI2 expression was significantly down regulated in TNBC tissues compared with normal tissues, in which MSI2a is the predominant functional isoform of MSI2 proteins in TNBC, as evident by the fact that overexpression of MSI2a inhibits TNBC cell invasion and extracellular signal regulated kinase 1/2 (ERK1/2) activity *in vitro* and *in vivo*. In addition, MSI2 directly regulates estrogen receptor 1 (ESR1) expression, which is a well-known therapeutic target, by binding to the specific sites in ESR1 RNA and increasing the stability of ESR1 protein, thus affecting the growth of breast cancer cells (47) **(** **Figure 8** **;** **Table 2** **)**.

## Conclusion

A large amount of evidence shows that the imbalance of RBPs occurs in various cancer types and affects every step of cancer development. With the progress of science and technology, new RBPs are constantly being reported, and the functions of RBPs will be enriched. Currently, the differential expression of many RBPs has been reported in breast cancer, which means that RBPs may become a new marker for tumor diagnosis and prognosis in the future. Meanwhile, inhibitors and compounds targeting the interaction between RBPs and target proteins are also emerging. As described in this review, small molecule inhibitor KH-3 and compound ZM-32 can inhibit the interaction between HuR and downstream mRNA, thus inhibiting the progression of breast cancer. Taken together, this review lists the roles of several RBPs and their target genes in the proliferation, cycle, apoptosis, migration and invasion of breast cancer cells. There are still few articles related to drug resistance, which can become a new research direction, and the complex regulatory network of RBP has not been fully understood. We need to have a more comprehensive understanding of the role of RBPs in breast cancer, which is expected to become a target for breast cancer therapy in the near future.

## Author contributions

All authors listed have made a substantial, direct, and intellectual contribution to the work and approved it for publication.

## Funding

This work was supported by the National Natural Science Foundation of China (82173842) and the Priority Academic Program Development (PAPD) of Jiangsu Higher Education Institutions. The presented figures were drawn by Figdraw.

## Conflict of interest

The authors declare that the research was conducted in the absence of any commercial or financial relationships that could be construed as a potential conflict of interest.

## Publisher’s note

All claims expressed in this article are solely those of the authors and do not necessarily represent those of their affiliated organizations, or those of the publisher, the editors and the reviewers. Any product that may be evaluated in this article, or claim that may be made by its manufacturer, is not guaranteed or endorsed by the publisher.
